# Aqua­bis­(4-chloro­benzoato)-κ^2^ *O*,*O*′;κ*O*-bis­(pyridine-κ*N*)cobalt(II)

**DOI:** 10.1107/S1600536813006752

**Published:** 2013-03-16

**Authors:** Ya-Li Chen, Chun-E Zhang, Peng Fei, Chao Deng, Bi-Tao Su

**Affiliations:** a Key Laboratory of Eco-Environment-Related Polymer Materials, Ministry of Education of China, Key Laboratory of Polymer Materials of Gansu Province, College of Chemistry and Chemical Engineering, Northwest Normal University, Lanzhou 730070, People’s Republic of China

## Abstract

In the title compound, [Co(C_7_H_4_ClO_2_)_2_(C_5_H_5_N)_2_(H_2_O)], the Co^II^ atom is six-coordinated by three O atoms from a bidentate and a monodentate 4-chloro­benzoate ligand, two N atoms from two pyridine ligands and a water O atom, giving a distorted octa­hedral geometry. In the crystal, the complex mol­ecules are connected by O—H⋯O hydrogen bonds and π–π interactions between the benzene rings [centroid–centroid distance = 3.8924 (17) Å] into a chain along [010]. Between adjacent chains, π–π inter­actions occur between the pyridine rings [centroid–centroid distance = 3.898 (2) Å], giving an overall two-dimensional architecture.

## Related literature

For structures and applications of related compounds, see: Macgillivray *et al.* (1998 ▶); Masaoka *et al.* (2001 ▶); Qiu *et al.* (2008 ▶); Wang & Sun (2012 ▶).
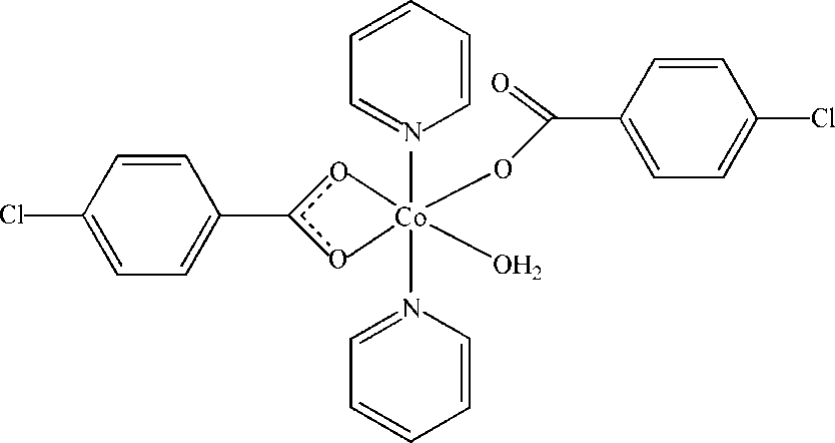


## Experimental

### 

#### Crystal data

[Co(C_7_H_4_ClO_2_)_2_(C_5_H_5_N)_2_(H_2_O)]
*M* *_r_* = 546.25
Monoclinic, 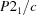

*a* = 15.1157 (8) Å
*b* = 5.8696 (3) Å
*c* = 28.5419 (9) Å
β = 109.682 (3)°
*V* = 2384.4 (2) Å^3^
*Z* = 4
Mo *K*α radiation
μ = 0.98 mm^−1^
*T* = 298 K
0.40 × 0.30 × 0.20 mm

#### Data collection

Bruker APEXII CCD diffractometer
Absorption correction: multi-scan (*SADABS*; Sheldrick, 1996 ▶) *T*_min_ = 0.695, *T*_max_ = 0.828
15626 measured reflections
4175 independent reflections
3590 reflections with *I* > 2σ(*I*)
*R*_int_ = 0.028

#### Refinement

*R*[*F* ^2^ > 2σ(*F* ^2^)] = 0.039
*wR*(*F* ^2^) = 0.082
*S* = 1.10
4175 reflections
307 parameters
H-atom parameters constrained
Δρ_max_ = 0.48 e Å^−3^
Δρ_min_ = −0.43 e Å^−3^

### 

Data collection: *APEX2* (Bruker, 2007 ▶); cell refinement: *SAINT* (Bruker, 2007 ▶); data reduction: *SAINT*; program(s) used to solve structure: *SHELXS97* (Sheldrick, 2008 ▶); program(s) used to refine structure: *SHELXL97* (Sheldrick, 2008 ▶); molecular graphics: *SHELXTL* (Sheldrick, 2008 ▶); software used to prepare material for publication: *SHELXTL*.

## Supplementary Material

Crystal structure: contains datablock(s) I, global. DOI: 10.1107/S1600536813006752/hy2620sup1.cif

Structure factors: contains datablock(s) I. DOI: 10.1107/S1600536813006752/hy2620Isup2.hkl

Additional supplementary materials:  crystallographic information; 3D view; checkCIF report

## Figures and Tables

**Table 1:** Hydrogen-bond geometry (Å, °)

*D*—H⋯*A*	*D*—H	H⋯*A*	*D*⋯*A*	*D*—H⋯*A*
O5—H1*W*⋯O4^i^	0.84	1.81	2.648 (3)	178
O5—H2*W*⋯O2^i^	0.81	1.99	2.737 (3)	154

Symmetry code: (i) 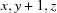.

**Fig. 1.**
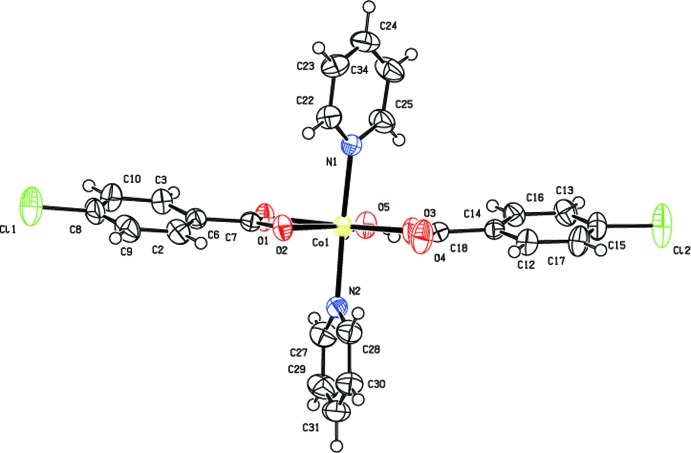
The molecular structure of the title complex. Displacement ellipsoids are drawn at the 30% probability level.

**Fig. 2.**
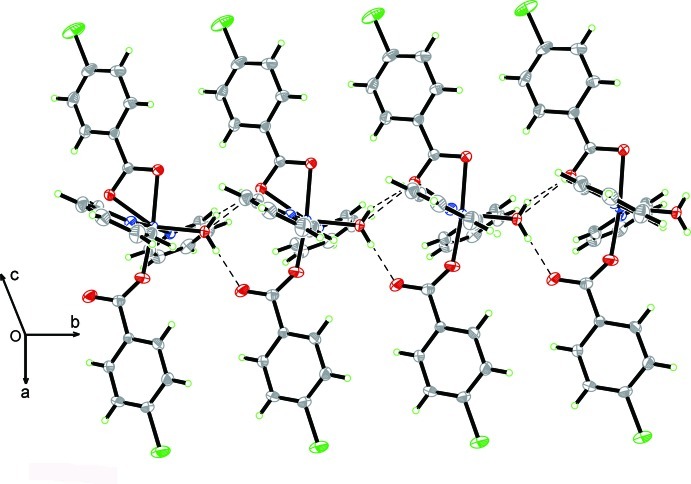
The chain structure of the title complex, constructed by hydrogen bonds (dashed lines).

## supplementary crystallographic information

### Comment

Metal-organic frameworks (MOFs) of aromatic acid are of great interest not only owing to their various structural motifs, but also due to their potential applications in the areas of material chemistry, medical chemistry, biological chemistry, molecular recognition and molecular device (Macgillivray *et al.*, 1998; Masaoka *et al.*, 2001). Sometimes hydrogen bonds play important roles in MOFs (Qiu *et al.*, 2008; Wang & Sun, 2012). In order to achieve MOFs by self-assembly and to explore their hydrogen bonds, herein we report the synthesis and crystal structure of a cobalt complex with 4-chlorobenzoic acid.

As shown in Fig. 1, the Co^II^ atom is six-coordinated by three O atoms from two 4-chlorobenzoate ligands, two N atoms from two pyridine molecules and a water molecule. The Co^II^ atom adopts a distorted octahedral geometry, in which two N atoms occupy the axial sites. The axial Co—N bond distances are 2.158 (2) and 2.168 (2) Å. The Co1—O3 bond distance [1.9931 (19) Å] for the monodentate 4-chlorobenzoate ligand is slightly shorter than Co1—O1 and Co1—O2 [2.1674 (18) and 2.2196 (18) Å] for the bidentate ligand. The crystal packing shows that the molecules are linked by O—H···O hydrogen bonds and π–π interactions between the benzene rings [centroid–centroid distances = 3.8924 (17) Å] into a polymeric chain along [010], as shown in Fig. 2. Between the adjacent chains, π–π interactions exist between the pyridine rings [centroid–centroid distance is 3.898 (2) Å] to give an overall 2D architecture.

### Experimental

A pyridine solution (5 ml) of Co(NO_3_)_2_.4H_2_O (0.1 mmol) was added dropwise to an ethyl acetate solution (15 ml) of 4-chlorobenzoic acid (0.2 mmol). The mixture was sealed in a Teflon-lined autoclave and heated under autogenous pressure to 120°C for 3 days and then allowed to cool to room temperature at a rate of 1°C per minute. Block-shaped purple crystals of the title complex were collected in 43% yield.

### Refinement

H atoms on C atoms were place at calculated positions and refined as riding atoms, with C—H = 0.93 Å and with *U*_iso_(H) = 1.2*U*_eq_(C). H atoms of water molecule were located from a difference Fourier map and refined as riding with *U*_iso_(H) = 1.5*U*_eq_(O).

### Crystal data

**Table:**

[Co(C_7_H_4_ClO_2_)_2_(C_5_H_5_N)_2_(H_2_O)]	*F*(000) = 1116
*M**_r_* = 546.25	*D*_x_ = 1.522 Mg m^−^^3^
Monoclinic, *P*2_1_/*c*	Mo *K*α radiation, λ = 0.71073 Å
Hall symbol: -P 2ybc	Cell parameters from 6935 reflections
*a* = 15.1157 (8) Å	θ = 2.4–28.2°
*b* = 5.8696 (3) Å	µ = 0.98 mm^−^^1^
*c* = 28.5419 (9) Å	*T* = 298 K
β = 109.682 (3)°	Block, purple
*V* = 2384.4 (2) Å^3^	0.40 × 0.30 × 0.20 mm
*Z* = 4	

### Data collection

**Table:**

Bruker APEXII CCD diffractometer	4175 independent reflections
Radiation source: fine-focus sealed tube	3590 reflections with *I* > 2σ(*I*)
Graphite monochromator	*R*_int_ = 0.028
φ and ω scans	θ_max_ = 25.0°, θ_min_ = 2.4°
Absorption correction: multi-scan (*SADABS*; Sheldrick, 1996)	*h* = −17→17
*T*_min_ = 0.695, *T*_max_ = 0.828	*k* = −6→6
15626 measured reflections	*l* = −33→33

### Refinement

**Table:**

Refinement on *F*^2^	Primary atom site location: structure-invariant direct methods
Least-squares matrix: full	Secondary atom site location: difference Fourier map
*R*[*F*^2^ > 2σ(*F*^2^)] = 0.039	Hydrogen site location: inferred from neighbouring sites
*wR*(*F*^2^) = 0.082	H-atom parameters constrained
*S* = 1.10	*w* = 1/[σ^2^(*F*_o_^2^) + (0.0201*P*)^2^ + 2.9673*P*] where *P* = (*F*_o_^2^ + 2*F*_c_^2^)/3
4175 reflections	(Δ/σ)_max_ = 0.001
307 parameters	Δρ_max_ = 0.48 e Å^−^^3^
0 restraints	Δρ_min_ = −0.43 e Å^−^^3^

### Special details

**Table:**

Geometry. All e.s.d.'s (except the e.s.d. in the dihedral angle between two l.s. planes) are estimated using the full covariance matrix. The cell e.s.d.'s are taken into account individually in the estimation of e.s.d.'s in distances, angles and torsion angles; correlations between e.s.d.'s in cell parameters are only used when they are defined by crystal symmetry. An approximate (isotropic) treatment of cell e.s.d.'s is used for estimating e.s.d.'s involving l.s. planes.
Refinement. Refinement of *F*^2^ against ALL reflections. The weighted *R*-factor *wR* and goodness of fit *S* are based on *F*^2^, conventional *R*-factors *R* are based on *F*, with *F* set to zero for negative *F*^2^. The threshold expression of *F*^2^ > σ(*F*^2^) is used only for calculating *R*-factors(gt) *etc*. and is not relevant to the choice of reflections for refinement. *R*-factors based on *F*^2^ are statistically about twice as large as those based on *F*, and *R*- factors based on ALL data will be even larger.

### Fractional atomic coordinates and isotropic or equivalent isotropic displacement parameters (Å^2^)

**Table:**

	*x*	*y*	*z*	*U*_iso_*/*U*_eq_	
Co1	0.23784 (2)	0.86593 (6)	0.875872 (13)	0.02636 (11)	
Cl1	−0.21542 (8)	0.3351 (2)	0.95560 (5)	0.0979 (4)	
Cl2	0.71492 (7)	0.92030 (19)	0.78128 (5)	0.0839 (4)	
O1	0.10651 (13)	0.9072 (3)	0.88994 (7)	0.0379 (5)	
O2	0.17142 (13)	0.5694 (3)	0.89912 (7)	0.0359 (5)	
O3	0.35454 (13)	0.7911 (4)	0.86129 (7)	0.0451 (5)	
O4	0.40222 (15)	0.4307 (4)	0.86863 (9)	0.0556 (6)	
O5	0.24608 (13)	1.2116 (3)	0.86343 (7)	0.0366 (5)	
H1W	0.2964	1.2776	0.8651	0.055*	
H2W	0.2166	1.2839	0.8774	0.055*	
N1	0.16311 (15)	0.8021 (4)	0.79767 (8)	0.0326 (5)	
N2	0.31910 (15)	0.9057 (4)	0.95367 (8)	0.0325 (5)	
C2	0.0318 (2)	0.3927 (5)	0.93584 (10)	0.0412 (7)	
H2	0.0852	0.3037	0.9411	0.049*	
C3	−0.0535 (2)	0.7357 (6)	0.90731 (11)	0.0420 (7)	
H3	−0.0569	0.8806	0.8936	0.050*	
C6	0.02714 (19)	0.6065 (5)	0.91523 (9)	0.0319 (6)	
C7	0.10590 (18)	0.7004 (5)	0.90078 (9)	0.0301 (6)	
C8	−0.1215 (2)	0.4410 (6)	0.94008 (12)	0.0524 (9)	
C9	−0.0427 (2)	0.3096 (6)	0.94881 (12)	0.0496 (8)	
H9	−0.0391	0.1663	0.9633	0.060*	
C10	−0.1284 (2)	0.6540 (6)	0.91935 (12)	0.0526 (9)	
H10	−0.1824	0.7411	0.9136	0.063*	
C12	0.55779 (19)	0.5577 (5)	0.84130 (11)	0.0374 (7)	
H12	0.5598	0.4156	0.8560	0.045*	
C13	0.5506 (2)	0.9779 (5)	0.79653 (11)	0.0420 (7)	
H13	0.5482	1.1187	0.7812	0.050*	
C14	0.48302 (18)	0.7019 (4)	0.83676 (9)	0.0288 (6)	
C15	0.6245 (2)	0.8322 (5)	0.80206 (12)	0.0436 (7)	
C16	0.4800 (2)	0.9117 (5)	0.81416 (10)	0.0356 (7)	
H16	0.4298	1.0094	0.8108	0.043*	
C17	0.6296 (2)	0.6223 (5)	0.82424 (12)	0.0471 (8)	
H17	0.6802	0.5258	0.8277	0.056*	
C18	0.40645 (18)	0.6316 (5)	0.85674 (10)	0.0343 (6)	
C22	0.1111 (2)	0.6170 (5)	0.78027 (11)	0.0402 (7)	
H22	0.1064	0.5078	0.8029	0.048*	
C23	0.0641 (2)	0.5797 (5)	0.73044 (11)	0.0466 (8)	
H23	0.0285	0.4483	0.7200	0.056*	
C24	0.0700 (2)	0.7357 (6)	0.69669 (11)	0.0487 (8)	
H24	0.0374	0.7157	0.6629	0.058*	
C25	0.1706 (2)	0.9504 (6)	0.76401 (11)	0.0468 (8)	
H25	0.2081	1.0784	0.7750	0.056*	
C27	0.3169 (2)	1.0861 (5)	0.98112 (11)	0.0455 (8)	
H27	0.2752	1.2033	0.9667	0.055*	
C28	0.3784 (2)	0.7390 (5)	0.97579 (11)	0.0425 (7)	
H28	0.3801	0.6097	0.9573	0.051*	
C29	0.3737 (3)	1.1083 (6)	1.03022 (12)	0.0572 (9)	
H29	0.3704	1.2379	1.0483	0.069*	
C30	0.4374 (2)	0.7476 (6)	1.02459 (11)	0.0497 (8)	
H30	0.4778	0.6275	1.0386	0.060*	
C31	0.4349 (2)	0.9365 (6)	1.05170 (12)	0.0545 (9)	
H31	0.4745	0.9486	1.0846	0.065*	
C34	0.1252 (2)	0.9234 (6)	0.71350 (11)	0.0541 (9)	
H34	0.1321	1.0317	0.6912	0.065*	

### Atomic displacement parameters (Å^2^)

**Table:**

	*U*^11^	*U*^22^	*U*^33^	*U*^12^	*U*^13^	*U*^23^
Co1	0.02674 (19)	0.02380 (19)	0.02987 (19)	−0.00091 (15)	0.01127 (14)	0.00061 (15)
Cl1	0.0735 (7)	0.1326 (11)	0.1053 (9)	−0.0426 (7)	0.0536 (7)	0.0101 (8)
Cl2	0.0694 (6)	0.0799 (7)	0.1319 (10)	−0.0037 (5)	0.0727 (7)	0.0154 (7)
O1	0.0372 (11)	0.0302 (11)	0.0534 (12)	0.0000 (9)	0.0246 (10)	0.0037 (9)
O2	0.0396 (11)	0.0296 (10)	0.0458 (11)	−0.0011 (9)	0.0240 (9)	−0.0020 (9)
O3	0.0355 (11)	0.0561 (14)	0.0504 (13)	0.0067 (10)	0.0233 (10)	0.0017 (11)
O4	0.0485 (13)	0.0444 (14)	0.0834 (17)	−0.0051 (11)	0.0348 (12)	0.0148 (12)
O5	0.0400 (11)	0.0224 (10)	0.0523 (13)	−0.0030 (8)	0.0220 (10)	−0.0017 (9)
N1	0.0320 (12)	0.0334 (13)	0.0325 (13)	−0.0028 (10)	0.0111 (10)	−0.0001 (10)
N2	0.0337 (12)	0.0308 (13)	0.0336 (12)	−0.0027 (10)	0.0120 (10)	−0.0015 (10)
C2	0.0516 (18)	0.0354 (16)	0.0411 (17)	−0.0053 (14)	0.0215 (14)	−0.0031 (14)
C3	0.0391 (17)	0.0479 (18)	0.0434 (18)	−0.0028 (14)	0.0195 (14)	0.0011 (15)
C6	0.0359 (15)	0.0320 (15)	0.0290 (14)	−0.0078 (12)	0.0127 (12)	−0.0058 (12)
C7	0.0316 (15)	0.0320 (16)	0.0268 (14)	−0.0024 (12)	0.0101 (12)	−0.0047 (12)
C8	0.051 (2)	0.069 (2)	0.0457 (19)	−0.0261 (18)	0.0284 (16)	−0.0083 (17)
C9	0.068 (2)	0.0407 (18)	0.0465 (19)	−0.0185 (17)	0.0278 (17)	−0.0013 (15)
C10	0.0381 (18)	0.069 (2)	0.057 (2)	−0.0014 (17)	0.0235 (16)	−0.0030 (18)
C12	0.0384 (16)	0.0290 (15)	0.0486 (18)	0.0013 (12)	0.0196 (14)	0.0022 (13)
C13	0.0524 (19)	0.0335 (16)	0.0463 (18)	−0.0039 (14)	0.0249 (15)	0.0013 (14)
C14	0.0287 (14)	0.0283 (14)	0.0296 (14)	−0.0035 (11)	0.0102 (11)	−0.0050 (11)
C15	0.0393 (17)	0.0460 (19)	0.0549 (19)	−0.0064 (14)	0.0283 (15)	−0.0045 (15)
C16	0.0383 (16)	0.0343 (16)	0.0375 (15)	0.0082 (13)	0.0169 (13)	0.0028 (13)
C17	0.0364 (17)	0.0445 (18)	0.068 (2)	0.0080 (14)	0.0280 (16)	0.0014 (17)
C18	0.0287 (14)	0.0429 (17)	0.0294 (14)	−0.0012 (13)	0.0075 (12)	−0.0009 (13)
C22	0.0436 (17)	0.0338 (16)	0.0405 (16)	−0.0031 (14)	0.0106 (13)	0.0024 (14)
C23	0.0450 (18)	0.0433 (19)	0.0443 (18)	−0.0075 (15)	0.0055 (15)	−0.0102 (15)
C24	0.054 (2)	0.056 (2)	0.0304 (17)	0.0062 (17)	0.0064 (15)	−0.0060 (15)
C25	0.059 (2)	0.0460 (18)	0.0364 (17)	−0.0149 (16)	0.0173 (15)	0.0008 (14)
C27	0.0542 (19)	0.0370 (18)	0.0449 (18)	−0.0008 (14)	0.0161 (15)	−0.0032 (14)
C28	0.0462 (18)	0.0401 (17)	0.0403 (18)	0.0021 (15)	0.0135 (14)	−0.0021 (14)
C29	0.080 (3)	0.050 (2)	0.0416 (19)	−0.018 (2)	0.0209 (18)	−0.0157 (17)
C30	0.0466 (19)	0.060 (2)	0.0375 (18)	0.0030 (16)	0.0071 (15)	0.0085 (16)
C31	0.056 (2)	0.069 (2)	0.0323 (17)	−0.0182 (19)	0.0068 (15)	0.0001 (17)
C34	0.075 (2)	0.054 (2)	0.0351 (17)	−0.0041 (18)	0.0209 (17)	0.0077 (15)

### Geometric parameters (Å, º)

**Table:**

Co1—O3	1.9931 (19)	C12—C14	1.382 (4)
Co1—O5	2.0710 (18)	C12—C17	1.384 (4)
Co1—N2	2.158 (2)	C12—H12	0.9300
Co1—O1	2.1674 (18)	C13—C15	1.373 (4)
Co1—N1	2.168 (2)	C13—C16	1.381 (4)
Co1—O2	2.2196 (18)	C13—H13	0.9300
Cl1—C8	1.738 (3)	C14—C16	1.384 (4)
Cl2—C15	1.741 (3)	C14—C18	1.510 (4)
O1—C7	1.253 (3)	C15—C17	1.376 (4)
O2—C7	1.268 (3)	C16—H16	0.9300
O3—C18	1.256 (3)	C17—H17	0.9300
O4—C18	1.235 (4)	C22—C23	1.377 (4)
O5—H1W	0.8405	C22—H22	0.9300
O5—H2W	0.8101	C23—C24	1.354 (4)
N1—C25	1.329 (4)	C23—H23	0.9300
N1—C22	1.335 (4)	C24—C34	1.368 (5)
N2—C27	1.324 (4)	C24—H24	0.9300
N2—C28	1.334 (4)	C25—C34	1.381 (4)
C2—C6	1.378 (4)	C25—H25	0.9300
C2—C9	1.387 (4)	C27—C29	1.381 (4)
C2—H2	0.9300	C27—H27	0.9300
C3—C10	1.375 (4)	C28—C30	1.379 (4)
C3—C6	1.388 (4)	C28—H28	0.9300
C3—H3	0.9300	C29—C31	1.366 (5)
C6—C7	1.491 (4)	C29—H29	0.9300
C8—C9	1.370 (5)	C30—C31	1.359 (5)
C8—C10	1.372 (5)	C30—H30	0.9300
C9—H9	0.9300	C31—H31	0.9300
C10—H10	0.9300	C34—H34	0.9300
			
O3—Co1—O5	94.10 (8)	C17—C12—H12	119.6
O3—Co1—N2	90.09 (8)	C15—C13—C16	118.8 (3)
O5—Co1—N2	91.37 (8)	C15—C13—H13	120.6
O3—Co1—O1	173.52 (9)	C16—C13—H13	120.6
O5—Co1—O1	91.95 (7)	C12—C14—C16	119.1 (2)
N2—Co1—O1	92.10 (8)	C12—C14—C18	120.3 (2)
O3—Co1—N1	86.43 (8)	C16—C14—C18	120.7 (2)
O5—Co1—N1	91.91 (8)	C13—C15—C17	121.9 (3)
N2—Co1—N1	175.38 (9)	C13—C15—Cl2	118.5 (2)
O1—Co1—N1	91.03 (8)	C17—C15—Cl2	119.6 (2)
O3—Co1—O2	114.26 (8)	C13—C16—C14	120.8 (3)
O5—Co1—O2	151.53 (7)	C13—C16—H16	119.6
N2—Co1—O2	86.29 (8)	C14—C16—H16	119.6
O1—Co1—O2	59.84 (7)	C15—C17—C12	118.6 (3)
N1—Co1—O2	92.35 (8)	C15—C17—H17	120.7
C7—O1—Co1	91.20 (16)	C12—C17—H17	120.7
C7—O2—Co1	88.45 (16)	O4—C18—O3	126.3 (3)
C18—O3—Co1	144.4 (2)	O4—C18—C14	118.8 (3)
Co1—O5—H1W	123.2	O3—C18—C14	114.9 (3)
Co1—O5—H2W	110.7	N1—C22—C23	123.2 (3)
H1W—O5—H2W	111.6	N1—C22—H22	118.4
C25—N1—C22	116.5 (2)	C23—C22—H22	118.4
C25—N1—Co1	119.32 (19)	C24—C23—C22	119.5 (3)
C22—N1—Co1	124.12 (19)	C24—C23—H23	120.2
C27—N2—C28	117.0 (3)	C22—C23—H23	120.2
C27—N2—Co1	125.2 (2)	C23—C24—C34	118.3 (3)
C28—N2—Co1	117.81 (19)	C23—C24—H24	120.9
C6—C2—C9	120.3 (3)	C34—C24—H24	120.9
C6—C2—H2	119.8	N1—C25—C34	123.1 (3)
C9—C2—H2	119.8	N1—C25—H25	118.4
C10—C3—C6	121.3 (3)	C34—C25—H25	118.4
C10—C3—H3	119.3	N2—C27—C29	123.0 (3)
C6—C3—H3	119.3	N2—C27—H27	118.5
C2—C6—C3	118.9 (3)	C29—C27—H27	118.5
C2—C6—C7	121.6 (3)	N2—C28—C30	123.7 (3)
C3—C6—C7	119.5 (3)	N2—C28—H28	118.2
O1—C7—O2	120.5 (2)	C30—C28—H28	118.2
O1—C7—C6	120.0 (2)	C31—C29—C27	118.8 (3)
O2—C7—C6	119.5 (2)	C31—C29—H29	120.6
C9—C8—C10	121.8 (3)	C27—C29—H29	120.6
C9—C8—Cl1	119.2 (3)	C31—C30—C28	118.2 (3)
C10—C8—Cl1	119.1 (3)	C31—C30—H30	120.9
C8—C9—C2	119.2 (3)	C28—C30—H30	120.9
C8—C9—H9	120.4	C30—C31—C29	119.4 (3)
C2—C9—H9	120.4	C30—C31—H31	120.3
C8—C10—C3	118.4 (3)	C29—C31—H31	120.3
C8—C10—H10	120.8	C24—C34—C25	119.2 (3)
C3—C10—H10	120.8	C24—C34—H34	120.4
C14—C12—C17	120.9 (3)	C25—C34—H34	120.4
C14—C12—H12	119.6		

### Hydrogen-bond geometry (Å, º)

**Table:**

*D*—H···*A*	*D*—H	H···*A*	*D*···*A*	*D*—H···*A*
O5—H1*W*···O4^i^	0.84	1.81	2.648 (3)	178
O5—H2*W*···O2^i^	0.81	1.99	2.737 (3)	154

Symmetry code: (i) *x*, *y*+1, *z*.
